# Polyphyllin G induce apoptosis and autophagy in human nasopharyngeal cancer cells by modulation of AKT and mitogen-activated protein kinase pathways in vitro and in vivo

**DOI:** 10.18632/oncotarget.11839

**Published:** 2016-09-02

**Authors:** Jui-Chieh Chen, Ming-Ju Hsieh, Chih-Jung Chen, Jen-Tsun Lin, Yu-Sheng Lo, Yi-Ching Chuang, Su-Yu Chien, Mu-Kuan Chen

**Affiliations:** ^1^ Department of Biochemical Science and Technology, National Chiayi University, Chiayi, Taiwan; ^2^ Cancer Research Center, Changhua Christian Hospital, Changhua, Taiwan; ^3^ School of Optometry, Chung Shan Medical University, Taichung, Taiwan; ^4^ Graduate Institute of Biomedical Sciences, China Medical University, Taichung, Taiwan; ^5^ Department of Surgical Pathology, Changhua Christian Hospital, Changhua, Taiwan; ^6^ Department of Medical Technology, Jen-Teh Junior College of Medicine, Nursing and Management, Miaoli, Taiwan; ^7^ School of Medicine, Chung Shan Medical University, Taichung, Taiwan; ^8^ Hematology & Oncology, Changhua Christian Hospital, Changhua, Taiwan; ^9^ Department of Pharmacy, Changhua Christian Hospital, Changhua, Taiwan; ^10^ College of Health Sciences, Chang Jung Christian University, Tainan, Taiwan; ^11^ Center for General Education, Mingdao University, Changhua, Taiwan; ^12^ Department of Otorhinolaryngology-Head and Neck Surgery, Changhua Christian Hospital, Changhua, Taiwan

**Keywords:** Polyphyllin G, nasopharyngeal carcinoma, apoptosis, autophagy, MAPK

## Abstract

Polyphyllin G (also call polyphyllin VII), extract from rhizomes of *Paris yunnanensis Franch*, has been demonstrated to have strong anticancer activities in a wide variety of human cancer cell lines. Previous studies found that Polyphyllin G induced apoptotic cell death in human hepatoblastoma cancer and lung cancer cells. However, the underlying mechanisms of autophagy in human nasopharyngeal carcinoma (NPC) remain unclear. In this study, Polyphyllin G can potently induced apoptosis dependent on the activations of caspase-8, -3, and -9 and the changes of Bcl-2, Bcl-xL and Bax protein expression in different human NPC cell lines (HONE-1 and NPC-039). The amount of both LC3-II and Beclin-1 was intriguingly increased suggest that autophagy was induced in Polyphyllin G-treated NPC cells. To further clarify whether Polyphyllin G-induced apoptosis and autophagy depended on AKT/ERK/JNK/p38 MAPK signaling pathways, cells were combined treated with AKT inhibitor (LY294002), ERK1/2 inhibitor (U0126), p38 MAPK inhibitor (SB203580), or JNK inhibitor (SP600125). These results demonstrated that Polyphyllin G induced apoptosis in NPC cells through activation of ERK, while AKT, p38 MAPK and JNK were responsible for Polyphyllin G-induced autophagy. Finally, an administration of Polyphyllin G effectively suppressed the tumor growth in the NPC carcinoma xenograft model *in vivo*. In conclusion, our results reveal that Polyphyllin G inhibits cell viability and induces apoptosis and autophagy in NPC cancer cells, suggesting that Polyphyllin G is an attractive candidate for tumor therapies. Polyphyllin G may promise candidate for development of antitumor drugs targeting nasopharyngeal carcinoma.

## INTRODUCTION

Nasopharyngeal carcinoma (NPC) is a cancer that arises from the epithelium of nasopharynx. Although such cancer occurs rarely in Europe and America, its incidence is much higher in Africa and Southeast Asia [1]. Advanced staging is the most important prognostic factor worldwide in NPC, decreasing 5-year overall survival rate from 100%-86.1% for Stage I-II disease to 50.7% for Stage IV disease [2]. Its unique epidemiology, pathogenesis and relation to the Epstein–Barr virus make it different to other head and neck cancers [3]. Although the cancer is known for its good response to radiation therapy, chemotherapy still has its role in current protocol.

Apoptosis and autophagy are discrete cellular processes that are mediated by distinct groups of regulatory and executioner molecules [4, 5]. Apoptosis is type I form of programmed cell death that is executed by activated caspases, which are specific enzymes that participate in signaling cascades that culminate in the rapid removal of organelles and other cellular structures [6, 7]. Autophagy is a major intracellular degradation mechanism that operates under stress conditions to promote survival during starvation or to cause programmed type II cell death under specific conditions (e.g., apoptosis inhibition) [8–10]. Autophagy has been characterized as both a unique cell-death pathway and an adaptation to stress that promotes cell survival. [11]. Recently, extensive attention has been paid to the role of autophagy in cancer development and therapy [12–14]. The relationship between apoptosis and autophagy is complex, and varies according to cell types and stressors. It remains uncertain whether autophagy represents a mechanism for preventing apoptosis or for enacting non-apoptotic programmed cell death. Certain agents kill cancer cells through non-apoptotic pathways, and may circumvent chemo-resistance. Such agents may be promising for treating resistant cancers [15]. For this reason, tumor cells with apoptosis defects undergo autophagy, so inhibiting autophagy causes tumor cells to die through alternative mechanisms [16]. It has also been suggested that autophagy may provide a useful method to prevent cancer development, limit tumor progression, and enhance the efficacy of cancer treatments [17].

Many vegetables, fruits, and grains offer substantial protection against various cancers [18–20]. There is increasing focus on providing a scientific basis for using these agents as a preventive strategy for people with a high risk of cancer. Therefore, inducing the death of cancer cells using chemotherapeutic agents may help to achieve a successful chemotherapy in patients. *Rhizoma Paridis* mainly contains steroidal saponins, especially diosgenyl saponins and pennogenyl saponins [21]. Several steroidal saponins possessing anticancer properties against a variety of cancer cells have been isolated and identified from *Rhizoma Paridis* [22]. Polyphyllin G (also call polyphyllin VII), extract from rhizomes of *Paris yunnanensis Franch* [23–25]. Polyphyllin G has been demonstrated to have strong anticancer activities via suppression of proliferation, cell cycle arrest, and modulation of drug resistance in a wide variety of human cancer cell lines [24, 26–29]. Although Polyphyllin G exerts antiproliferative and proapoptotic activities in various cancer cell types, the underlying mechanisms of autophagy in human NPC remain unclear. The aim of this study was to characterize the effects of Polyphyllin G on human NPC cells, and elucidate the underlying molecular mechanism responsible for autophagy and apoptosis in Polyphyllin G-induced cytotoxicity.

## RESULTS

### Cytotoxic effects of Polyphyllin G on human NPC cell lines

**Figure 1:**
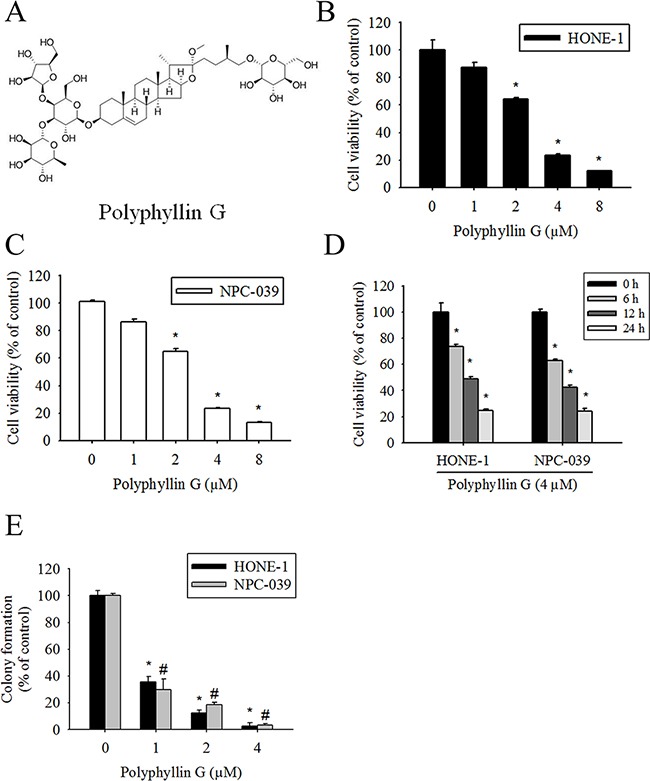
Polyphyllin G reduces cell viability in the dose- and time-dependent manners **A.** Chemical structure of Polyphyllin G. **B.** HONE-1 and **C.** NPC-039 cells were treated with indicated concentrations of Polyphyllin G for 24 h, after which the cell viability was measured using MTT assay. **D.** Cells cultured in the presence of Polyphyllin G (4 μM) for 6, 12 and 24 h, respectively. Cell viability was measured by MTT assay. Results are shown as mean ± SD from 3 determinations per condition repeated 3 times. *P < 0.05, compared with the control (0 μM or 0 h). **E.** Equal numbers of cells from the Polyphyllin G-treated HONE-1 and NPC-039 cell pools were plated and stained as described in the text. The number of colonies was counted under a dissecting microscope. The data show the relative colony number, and the number of cell lines without Polyphyllin G treatment was set at 100%. Results are shown as mean ± SE. *p < 0.05, compared with the HONE-1 (0 μM). #p < 0.05, compared with the NPC-039 (0 μM).

The chemical structure of Polyphyllin G is shown in Figure 1A. To assess the effects of Polyphyllin G on cell viability, HONE-1 and NPC-039 cells were cultured in the presence of increasing concentrations of Polyphyllin G for 24 h. As shown in Figure 1B-1C, Polyphyllin G significantly inhibited cell viability in a dose-dependent manner. Polyphyllin G (4 μM) also substantially decreased the cell viability of HONE-1 and NPC-039 cells in a time-dependent fashion, compared with untreated cells (Figure 1D). To further investigate the anti–cell-growth activity of Polyphyllin G, a clonogenic assay was performed to determine the long-term effect of Polyphyllin G treatment on NPC cancer cells. Polyphyllin G (25 μM) significantly inhibited the colony-formation ability of HONE-1 and NPC-039 cells (Figure 1E). These results indicated that Polyphyllin G can potently inhibit cell viability of different human NPC cell lines.

### Polyphyllin G-induced cell cycle arrest and cell apoptosis in human NPC cell lines

**Figure 2:**
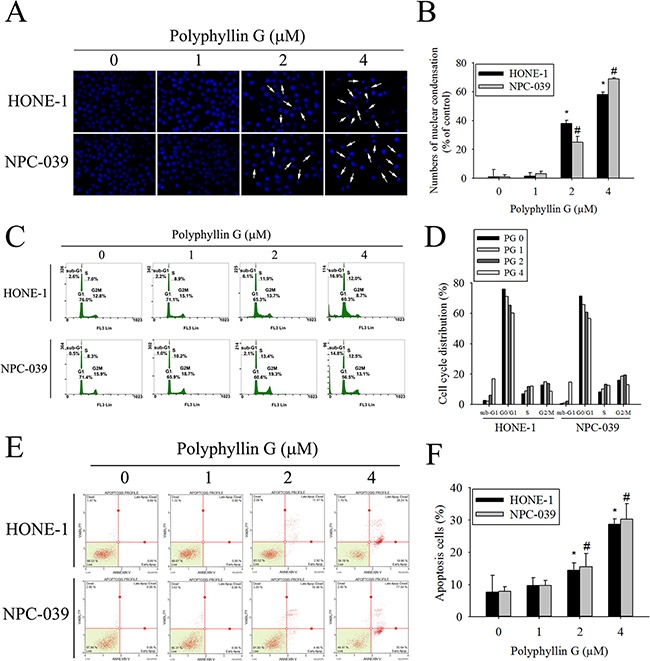
Polyphyllin G induces apoptosis in HONE-1 and NPC-039 cells **A.** Cells were treated with different concentration of Polyphyllin G (1-4 μM) for 24 h and then stained with DAPI. Fragmented or condensed nuclei could be observed under a fluorescence microscope as indicated by the arrows. **B.** Results are shown as mean ± SE from 3 determinations per condition repeated 3 times. *P < 0.05, compared with the control (0 μM). **C.** Cells were stained with propidium iodide (PI), and analyzed for DNA content by Muse Cell Analyzer flow cytometry. The percentages of apoptotic cells were evaluated by sub-G1 DNA content (hypodiploid DNA). **D.** The quantified data of cell cycle distribution. **E.** For quantitative analysis of apoptosis, cells were dual-labeled with PI and Annexin V fluorescence and analyzed by Muse Cell Analyzer flow cytometry. **F.** For quantitative analysis of apoptosis, cells were staining with Muse Caspase-3/7 assay kit and analyzed by Muse Cell Analyzer flow cytometry. Data are representative of at least 3 independent experiments. Results are shown as mean ± SE. *p < 0.05, compared with the HONE-1 (0 μM). #p < 0.05, compared with the NPC-039 (0 μM).

**Figure 3:**
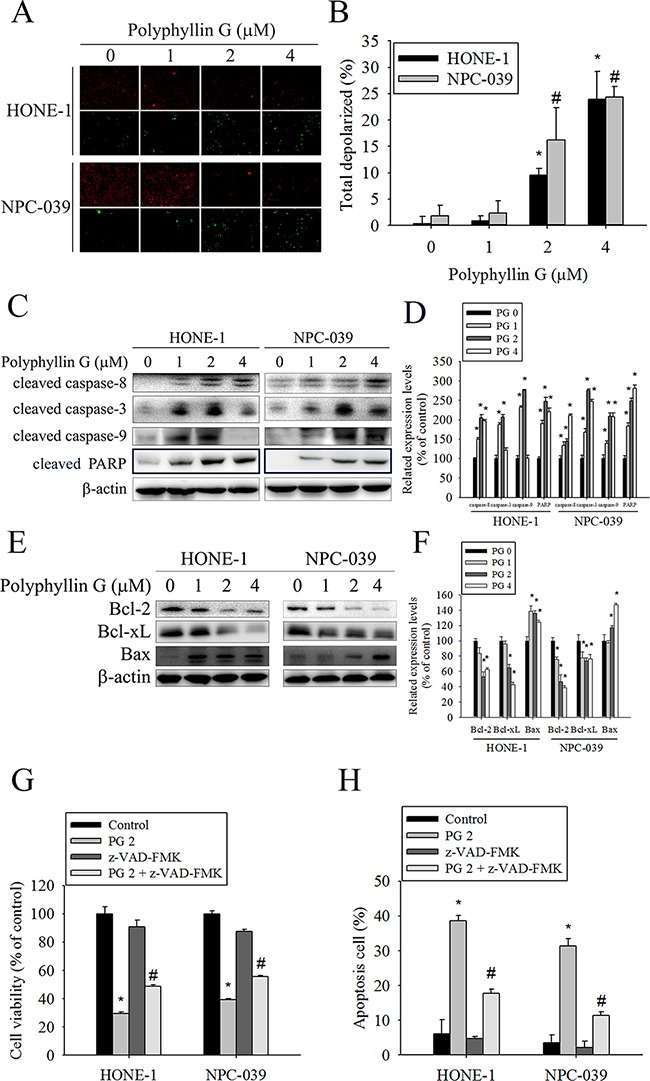
Effects of Polyphyllin G on apoptotic and anti-apoptotic protein expression in HONE-1 and NPC-039 cells **A.** Cells were treated with an indicated concentration of Polyphyllin G for 24 h and subjected to staining for JC-1. Fluorescent signals were observed using a fluorescence microscope. **B.** Cells were treated with increasing dose of Polyphyllin G for 24 h. The mitochondrial membrane potential was investigated by Muse Cell Analyzer flow cytometry and the data was processed by the Muse®Cell Analyzer Assays. **C.** A representative Western blot for expression of cleaved caspase-8, -3, -9 and PARP cleavage in cells after stimulation with Polyphyllin G at different concentration. **D.** The values under each lane indicate relative density of the band normalized to β-actin, respectively. **E.** Cells were treated with indicated concentration of Polyphyllin G for 24 h and subjected to Western blot with antibodies against Bcl-2, Bcl-xL-2, and Bax. **F.** Band intensity was quantified by densitometry analysis. β-actin was used as an internal control to normalize the amount of proteins applied in each lane. Values represent the mean ± SD of 3 independent experiments. *P < 0.05, compared with the control (0 μM). **G.** HONE-1 and NPC-039 cells (5 × 10^4^ cells/well of 24-well plate) were treated with Polyphyllin G (2 μM) in the presence or absence of apoptosis inhibitor z-VAD-FMK (20 μM) for 24 h, as analyzed by MTT assay and **H.** dual-labeled with PI and Annexin V fluorescence and analyzed by flow cytometry. Results are shown as mean ± SE. *P<0.05, compared with the control (0 μM). #P<0.05, compared with the Polyphyllin G treatment (2 μM).

To elucidate whether Polyphyllin G inhibits cell growth through the induction of apoptosis, we investigated the effects of Polyphyllin G on apoptosis in NPC cell lines. As shown in Figure 2A-2B, apoptotic cells with condensed and fragmented nuclei were gradually increased in a dose-dependent manner. We next analyzed cell cycle of Polyphyllin G-treated HONE-1 and NPC-039 cells. We observed a dose-dependent increase of the sub-G1 population, as assessed by flow cytometry (Figure 2C-2D). Furthermore, Annexin V/PI double staining and caspase-3/7 staining were also performed, and the results showed in a dose-dependent increase of both early and late apoptotic cells (Figure 2E-2F). To visualize the apoptotic features, cells were stained with JC-1. In the fluorescent images, a dose-dependent increase of green signal was detected in the cells treated with Polyphyllin G. The mitochondrial membrane potential was reduced in Polyphyllin G-treated NPC cell lines (Figure 3A-3B). To further elucidate the mechanisms of Polyphyllin G-induced apoptosis in NPC cells, we evaluated the involvement of apoptosis-related proteins in the apoptotic process by Western blot analysis. After the treatment with Polyphyllin G for 24 h, cleavage of caspase-8, caspase-3, caspase-9, and PARP significantly increased in a dose-dependent manner (Figure 3C-3D). Furthermore, Polyphyllin G also caused a dramatic dose-dependent decrease in the protein level of Bcl-2 and Bcl-xL, while Bax protein level was significantly increased (Figure 3E-3F). Collectively, these data demonstrated Polyphyllin G-induced apoptosis was dependent on the activations of caspase-8, -3, and -9 and the changes of Bcl-2, Bcl-xL and Bax protein expression. To clarify the relevance of Polyphyllin G-induced cell death, z-VAD-FMK (a broad-spectrum caspase inhibitor) was used in the following experiments. Polyphyllin G combined with Z-VAD-FMK substantially increase the cell viability and decrease apoptosis cells of HONE-1 and NPC-039 cells (Figure 3G-3H).

### Polyphyllin G induced autophagy via inhibition of mTOR phosphorylation in NPC cells

**Figure 4:**
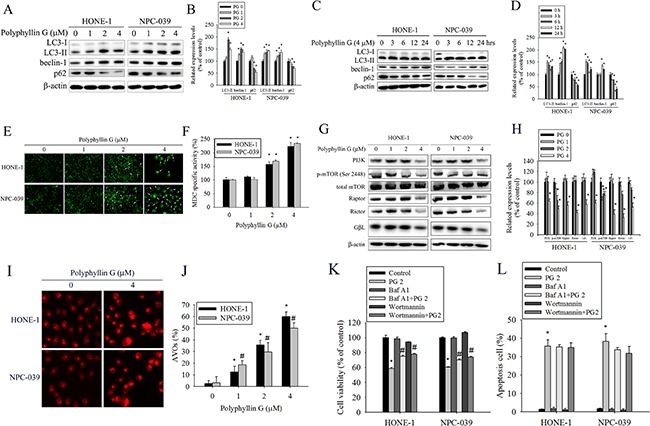
Polyphyllin G induces autophagy in HONE-1 and NPC-039 cells **A.** Cells were treated with indicated concentration of Polyphyllin G for 24 h and subjected to Western blot with antibodies against LC3-I/LC3-II, beclin-1, and p62. **B.** The intensity of bands was quantified by densitometric analysis. **C.** Cells were incubated with Polyphyllin G (1 μM) for the indicated time intervals and the expression levels of LC3-I/LC3-II, beclin-1, and p62 were examined by Western blot. **D.** The intensity of bands was quantified by densitometry analysis. **E.** Cells seeded onto glass coverslips with treatment with Polyphyllin G for 24 h, followed by immunostaining and an observation of LC-3 (green fluorescence) under fluorescence microscopy. Original magnifications: 200×. **F** Cells were treated for 24 h in the presence of Polyphyllin G (1-4 μM), and MDC was added to the medium during the last hour of culture. The quantification of MDC-stained structures was performed using the analyze particles tool of the Image J software on an average of 100 cells per treatment. The data show the mean ± SD of at least 3 independent experiments. *P < 0.05, compared with the control (0 μM). **G.** Cells were treated with indicated concentration of Polyphyllin G for 24 h and subjected to Western blot with antibodies against PI3K, mTOR (ser2448), mTOR, Raptor, Rictor and GβL. **H.** The intensity of bands was quantified by densitometric analysis. **I.** Cells treatment with Polyphyllin G for 24 h was stained with acridine orange for AVOs formation. Data shown are representative fluorescence microscopic photographs. Original magnifications: 200×. **J.** The quantification of AVOs, acridine orange-stained cells were harvested and then analyzed by flow cytometry. **K.** HONE-1 and NPC-039 cells (5 × 10^4^ cells/well of 24-well plate) were treated with Polyphyllin G (2 μM) in the presence or absence of autophagy inhibitor Baf A1 (1 nM) or wortmannin (50 nM) for 24 h, as analyzed by MTT assay and **L.** dual-labeled with PI and Annexin V fluorescence and analyzed by flow cytometry. Results are shown as mean ± SE. *P<0.05, compared with the control (0 μM). #P<0.05, compared with the Polyphyllin G treatment (2 μM).

Although a number of commonly used anticancer drugs have shown to induce apoptosis in cancer cells, autophagy is also frequently observed in response to these treatments [30, 31]. We next investigated whether Polyphyllin G induces autophagy in NPC cells. To further confirm the involvement of autophagy in Polyphyllin G treatment, we analyzed the expression of the autophagic markers LC3I/II, Beclin-1, and the cargo receptor p62 by Western blot. As shown in (Figure 4A-4D), the amount of both LC3-II and Beclin-1 was intriguingly increased in both tested cells. Additionally, Polyphyllin G significantly decreased the level of p62. These changes in the expression levels of autophagy-related proteins indicated enhanced level of autophagy in Polyphyllin G-treated NPC cells. After 24 h of treatment with Polyphyllin G, a substantial change in LC3 puncta formation was observed, indicating the formation of autophagosomes in Polyphyllin G-treated HONE-1 and NPC-039 cells (Figure 4E). To confirm autophagic induced by Polyphyllin G, MDC staining was performed and viewed under a fluorescence microscope. MDC is a specific marker for autophagic vacuoles. Similar to Western blots, MDC-labeled autophagic vacuoles appeared as distinct dot gradually increased in a dose-dependent manner (Figure 4F). The mTOR pathway functions as an autophagy regulator under starvation or other cellular stress conditions [31]. We detected the expression of PI3K, activated mTOR (ser2448), total mTOR, Raptor, Rictor and GβL in HONE-1 and NPC-039 cells treated with different concentrations of Polyphyllin G. As shown in Figure 4G-4H, Western blotting assays revealed that Polyphyllin G significantly decreased the expression of PI3K, activated mTOR (ser2448), total mTOR, Raptor, Rictor and GβL in a dose-dependent manner. Acidic vesicular organelle (AVO) formation (autophagosomes and autolysosomes) is a characteristic feature of autophagy [32] To detect the development of AVOs, untreated and treated HONE-1 and NPC-039 cells were stained with acridine orange. Polyphyllin G induced AVO formation in NPC cells (Figure 4I-4J). To clarify the relevance of Polyphyllin G-induced cell death, bafilomycin A1 (Baf A1) and wortmannin (autophagy inhibitor) was used in the following experiments. Polyphyllin G combined with Baf A1 and wortmannin substantially increase the cell viability and no effect apoptosis cells of only Polyphyllin G-treatment HONE-1 and NPC-039 cells (Figure 4K-4L).

### AKT/ERK/JNK/p38 MAPK signaling pathways are involved in Polyphyllin G-induced apoptosis and autophagy

**Figure 5:**
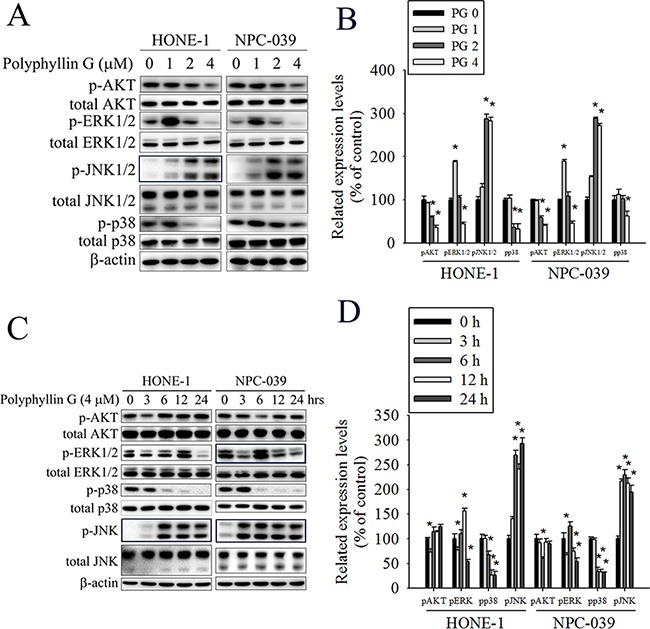
Polyphyllin G induces the phosphorylation of AKT, ERK1/2, JNK1/2, and p38 MAPK in HONE-1 and NPC-039 cells **A.** Cells were treated with different concentrations of Polyphyllin G (0-4 μM) for 24 h. The p-AKT, p-ERK1/2, p-JNK1/2, and p-p38 were analyzed by Western blot with their respective antibodies. The total protein of AKT, ERK1/2, JNK1/2, and p38 is also shown. **B.** Quantitative results of p-AKT, p-ERK1/2, p-JNK1/2, and p-p38 protein levels, which were adjusted with their total protein levels and expressed as multiples of induction beyond each respective control. **C.** Cells were incubated with Polyphyllin G (4 μM) for indicated time intervals and the phosphorylation status and total levels of proteins were measured by Western blot. **D.** Quantitative analyses of the phosphorylation status and total levels of proteins. Values represent the mean ± SD of at least 3 independent experiments. *P < 0.05, compared with the control (0 μM or 0 h).

**Figure 6:**
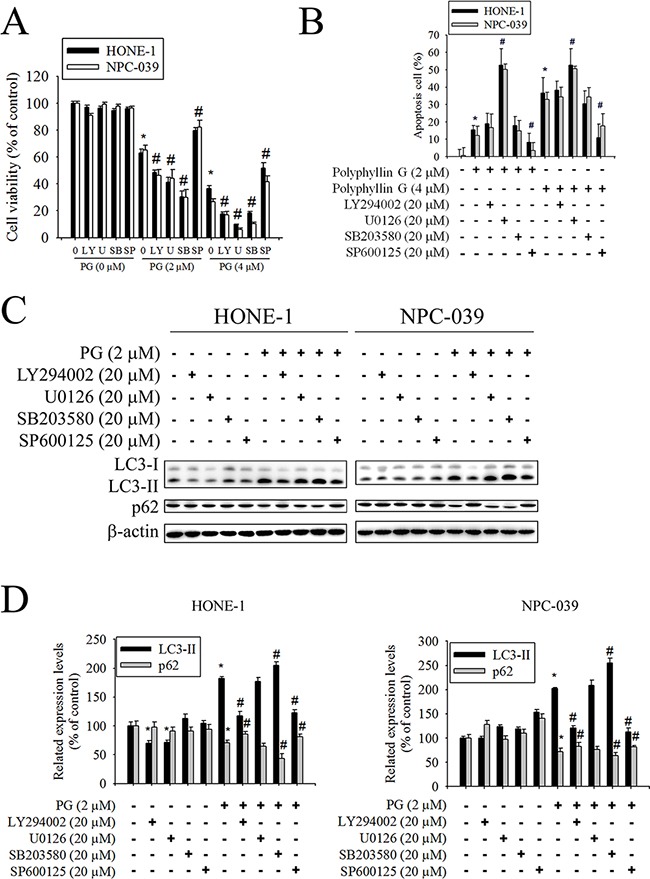
Effects of the AKT, ERK1/2, JNK1/2, and p38 MAPK on Polyphyllin G-induced apoptosis and autophagy in HONE-1 and NPC-039 cells Cells were pretreated with LY294002 (AKT inhibitor, 20 μM), U0126 (ERK1/2 inhibitor, 20 μM), SB203580 (p38 MAPK inhibitor, 20 μM), or SP600125 (JNK1/2 inhibitor, 20 μM) for 1 h followed by treatment with or without Polyphyllin G for 24 h. **A.** Cell viability was measured by MTT assay. **B.** The percentages of apoptotic cells were evaluated by flow cytometry. **C.** The levels of expression of LC3-I/LC3-II and p62 were examined by Western blot. **D.** Band intensity was quantified by densitometry analysis. β-actin was used as an internal control to normalize the amount of proteins applied in each lane. Values represent the mean ± SD of 3 independent experiments. *P<0.05 vs. control (cells treated only with DMSO), and #*P*<0.05 vs. Polyphyllin G.

Previously studies have reported that AKT and MAPKs signaling pathways are implicated in apoptosis and autophagy [33–36]. To further investigate the mechanism by which Polyphyllin G induces apoptosis and autophagy, we directly measured the phosphorylation of AKT and MAPKs in response to Polyphyllin G. The results revealed that stimulation of cells to Polyphyllin G induced an increase in phosphorylation of JNK1/2 in a dose-dependent manner (Figure 5A-5B). Moreover, treatment of NPC cells with Polyphyllin G (4 μM) also resulted in a time-dependent increase in phosphorylation of JNK (Figure 5C-5D). To further clarify whether Polyphyllin G-induced apoptosis and autophagy depended on AKT/ERK/JNK/p38 MAPK signaling pathways, cells were treated with AKT inhibitor (LY294002), ERK1/2 inhibitor (U0126), p38 MAPK inhibitor (SB203580), or JNK1/2 inhibitor (SP600125) for 2 h followed by treatment with or without Polyphyllin G for 24h. As shown in Figure 6A, cell viability of NPC cells that pretreated with SP600125 and then treated with Polyphyllin G was increased cell viability when compared with Polyphyllin G treatment only. However, the combination of LY294002, U0126 and SB203580 with Polyphyllin G dramatically decreased cell viability in NPC cells. In addition, pre-treatment with U0126 was increased Polyphyllin G-induced apoptosis. Pre-treatment with SP600125 was decreased Polyphyllin G-induced apoptosis. However, LY294002 or SB203580 does not affect apoptosis by Polyphyllin G. Suggesting the activation of ERK1/2 and JNK1/2 was involved in cell apoptosis (Figure 6B). To address the direct role of signaling in autophagy induction, the effects of pharmacological inhibitors on the proteins levels of LC3 and p62 were examined. Our date showed that Polyphyllin G-induced autophagy was dramatically decreased in Polyphyllin G co-treatment with LY294002 or SP600125 group compared with Polyphyllin G treatment alone (Figure 6C-6D). In addition, pre-treatment with SB203580 was increased Polyphyllin G-induced autophagy suggesting the activation of AKT, p38 MAPK or JNK1/2 was involved in cell autophagy. Taken together, these results demonstrated that Polyphyllin G induced apoptosis in NPC cells through ERK1/2 reduction and activation of JNK1/2, while AKT, p38 MAPK and JNK1/2 were responsible for Polyphyllin G-induced autophagy.

### Significant antiproliferative effects of Polyphyllin G in an NPC orthotopic graft model

**Figure 7:**
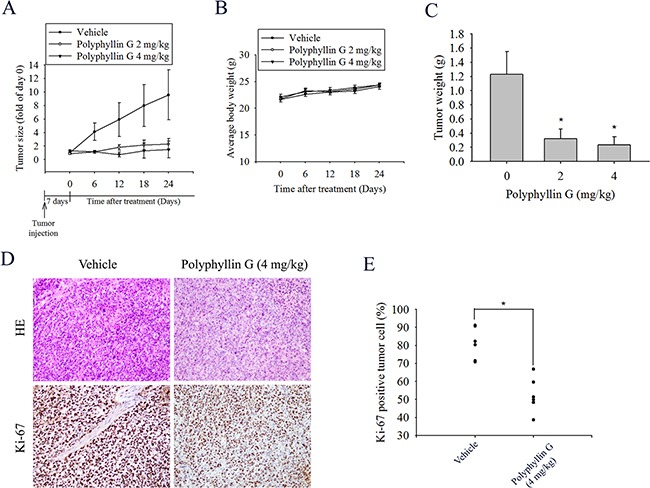
Polyphyllin G suppressed tumor growth NPC-039 cells *in vivo* **A.** NPC-039 cells were injected into the right flank of 6-week-old male nude mice. After injection of NPC-039 cells, nude mice were treated with either vehicle or Polyphyllin G (2 or 4 mg/kg) for 24 days. The growth of the xenograft tumors was referred to the measurement of the long and short dimensions of the tumors, and the calculation of the tumor size were described in the “Materials and methods” section. **B.** Body weight and **C.** tumor weight changes of the mice during 24 days of treatment. *P < 0.05, compared to the vehicle. **D.** Tumor tissues were examined by H&E staining, and immunohistochemical staining with an anti-Ki67 antibody. Original magnifications: 200×. **E.** The mean value of the Ki67 expression percentage is indicated by a red bar. *p < 0.05, compared to the vehicle groups.

To test the effect of Polyphyllin G on tumor growth, the in vivo antitumor effect of Polyphyllin G was evaluated. Tumor volumes were determined by caliper measurements for every 6 days. The control group of animals transplanting NPC-039 cancer cells showed a progressive increase in their tumor volumes. In Polyphyllin G-treated mice receiving 2 and 4 mg/kg, the mean tumor volume (Figure 7A) and tumor weight (Figure 7C) were significantly inhibited compared to vehicle-treated. As illustrated in Figure 7B, no significant difference in body weight was detected among these groups. In IHC analysis for cell proliferation, Ki67-positive tumor cells were significantly reduced after treatment with Polyphyllin G compared to control mice (Figure 7D-7E). These results showed that animals treated with Polyphyllin G show anti-tumor growth as compared with control animals.

## DISCUSSION

Polyphyllin G (also call Polyphyllin VII) exhibited anti-proliferative activity against the hepatocellular carcinoma HepG2 cell line [26, 37], MCF-7/ADR cell line [27] and lung cancer cells lines (A549 and NCI-H1299) [28]. The present study first investigated the significant *in vitro* and *in vivo* inhibitory effects of Polyphyllin G on human nasopharyngeal cancer cells. Our data demonstrated that Polyphyllin G inhibited the growth of HONE-1 and NPC-039 cells at low IC50 values in a dose-dependent manner (Table 1). These results suggest the potent anti-cancer effects of Polyphyllin G. Various natural chemicals have been reported to exhibit anticancer activity by triggering both autophagy and apoptosis [38–41], implicated the close relationship between autophagy and apoptosis. Polyphyllin G has been demonstrated to have strong anticancer activities in a wide variety of human cancer cell lines [24, 26–28]. However, the underlying mechanisms of Polyphyllin G-induced apoptosis and autophagy in human nasopharyngeal cancer cells is remain unclear.

**Table 1:** IC50 of Polyphyllin G in HONE-1 and NPC-039 cell lines for 24 h

IC50 (24 h)	Polyphyllin G (μM)
HONE-1	2.33±0.22
NPC-039	2.30±0.31

Previous studies show that Polyphyllin G arrested A549 and NCI-H1299 cells in the G2/M phase [28]; Li et al point that Polyphyllin G could induce cell apoptosis, together with cell cycle arrest in G1 phase, and trigger apoptosis in a caspase-3-dependent manner in human colorectal cancer cells (HT-29 and SW-620) [42]. In this study, the flow cytometric analysis of PI-labeled cells shows that treating NPC cell lines with Polyphyllin G (4 μM) induced significant accumulation of cells in the sub-G1 phase (Figure 2B). The sub-G1 cell population increased to 16.9% and 14.8% in HONE-1 and NPC-039 cells treated with 4 μM Polyphyllin G for 24 h, respectively. Moreover, Annexin V/PI double staining was also performed, and the results showed in a dose-dependent increase of both early and late apoptotic cells. Previous study show that Polyphyllin G increased TNFR1, TRAIL R1/DR4, TRAIL R2/DR5, and FADD expression, and activation of PARP, caspase-8, and 3 in MCF-7/ADR cells [43]. Zhang et al point that Polyphyllin G upregulated cleaved caspase-3, cleaved caspase-9, and Bax expression in Hela cells [44]. Polyphyllin G treatment reduced the total Bcl-2 levels in HepG2 cells [26]. Our data demonstrated Polyphyllin G-induced apoptosis was dependent on the activations of caspase-8, -3, and -9 and the changes of Bcl-2, Bcl-xL and Bax protein expression (Figure 3). These results demonstrated that apoptosis developed in NPC-039 and HONE-1 cells after they were treated with Polyphyllin G for 24 h, and that the extent of apoptosis were related to the dose of Polyphyllin G administrated.

Autophagy is a major intracellular degradation mechanism that operates under stress conditions to promote survival during starvation or to cause programmed type II cell death under specific conditions [8–10]. Autophagy is initiated when large sections of cytoplasm are engulfed by a crescent-shaped phagophore that elongates to form an autophagosome, which subsequently fuses with a lysosome, resulting in the degradation of cellular content by lysosomal hydrolases [16, 17]. Previous study point that Polyphyllin G induces an autophagic cell death in HepG2 cells via inhibition of PI3K/AKT/mTOR, and activation of JNK pathway, which induces phosphorylation of Bcl-2 and dissociation of Beclin-1 from Beclin-1/Bcl-2 complex, leading to induction of autophagy [26]. Polyphyllin G inhibited cell growth by down-regulating MEK1/2, ERK1/2 phosphorylation, and suppression of AKT pathway [42]. Chen et al show that Polyphyllin G was associated with apoptosis induction and proliferation inhibition through regulation of MAPK pathway and inhibition of PI3K/Akt pathway [37]. The present study demonstrated that Polyphyllin G induced apoptosis in NPC cells through activation of ERK, while AKT, p38 MAPK and JNK were responsible for Polyphyllin G-induced autophagy.

The present study demonstrated that Polyphyllin G suppressed the human nasopharyngeal cancer cells tumor formation in the xenograft model *in vivo* (Figure 7). The mean tumor volume in Polyphyllin G treated mice was smaller compared to control, however, is somewhat at odds with the *in vitro* data (Figure 1). Previous study show that Polyphyllin G inhibited the growth of lung cancer tumor xenografts at the dose of 1, 2, and 3 mg/kg [28]. Present study results are consistent with the previous study and further illustrated that Polyphyllin G may also be able to apply on different cancers. The assumption credibility need more research to further prove.

In conclusion, our results reveal that Polyphyllin G inhibits cell viability and induces apoptosis and autophagy in NPC cancer cells, suggesting that Polyphyllin G is an attractive candidate for tumor therapies. We believe that Polyphyllin G may promise candidate for development of antitumor drugs targeting nasopharyngeal carcinoma.

## MATERIALS AND METHODS

### Chemicals

Polyphyllin G (purity > 98%) was purchased from Santa Cruz Biotechnology (Santa Cruz, CA) and was dissolved in dimethyl sulfoxide (DMSO) and diluted in cell culture medium to the final concentration before use. The final concentration of DMSO for all treatments was consistently less than 0.1%. All cell culture-related reagents were purchased from Invitrogen (Carlsbad, CA). DAPI dye, propidium iodide (PI), RNase A, protease inhibitor cocktail, phosphatase inhibitor cocktail, and 3-(4, 5-dimethylthiazol-2-yl)-2, 5-diphenyltetrazolium bromide (MTT) were purchased from Sigma-Aldrich (St Louis, MO). Antibody against cleaved caspase-3, -8, and -9, cleaved poly (ADP-ribose) polymerase (PARP), Bcl-2, Bcl-xL, Bax, LC3B, Beclin1, p62, p-AKT, AKT, p-ERK1/2, ERK1/2, p-JNK1/2, JNK1/2, p-p38, p38, PI3K, mTOR (ser2448), mTOR, Raptor, Rictor, GβL and β-actin were purchased from Cell Signaling Technology (Danvers, MA). Specific inhibitors for AKT inhibitor (LY294002), ERK1/2 (U0126), p38 MAPK (SB203580), JNK (SP600125), z-VAD-FMK, Wortmannin and Bafilomycin A1 (Baf A1) were purchased from Santa Cruz Biotechnology (Santa Cruz, CA).

### Cell culture

The human nasopharyngeal carcinoma (NPC) cell lines HONE-1 and NPC-039 cells were a gift from Dr. Jen-Tsun Lin, Hematology & Oncology, Changhua Christian Hospital (Supplemental data 1). All cells were cultured in RPMI 1640Medium (Gibco BRL, Grand Island, NY) supplemented with10% fetal bovine serum (FBS), 0.1 mM non-essential amino acids,1 mM glutamine, 1% penicillin/streptomycin, 1.5 g/l sodium bicarbonate, and 1 mM sodium pyruvate (Sigma, St. Louis, MO) and maintained at 37°C in a humidified atmosphere of 5% CO_2_.

### Cell cytotoxicity

MTT assay was used to evaluate the effect of Polyphyllin G on cell viability. Briefly, cells were seeded into 96 well plates at a density of 5000 cells/well containing 100 μl of culture medium. After overnight incubation, the cells were treated with indicated Polyphyllin G concentration. At every indicated time interval after Polyphyllin G treatment, 10 μl MTT solution was added to each well (at a final concentration of 5 mg/ml) and incubated for further 4 h. The supernatant was then discarded, and 200 μl of DMSO (dimethyl sulfoxide) was added to each well to dissolve the formazan. Optical density (OD) was evaluated by measuring the absorbance, with a test wavelength of 490 nm and a reference wavelength of 630 nm.

### Colony formation

HONE-1 and NPC-039 cell line was seeded at a concentration of 0.05x10^4^ cells per well in 6-well cell culture plates in appropriate media. After 24 h, media was replaced with fresh media containing either Polyphyllin G at 1, 2 and 4 μM. Medium with compound being changes every 3 days. After 2 weeks, colonies were stained with 0.3 % crystal violet solution.

### Detection of apoptosis by DAPI staining

After being subjected to indicate treatment, cells were collected and fixed with 4% paraformaldehyde for 20 min. Then, cells painted on the slides and stained with DAPI dye (50 μg/ml) for 10 min. After washing with phosphate-buffered saline (PBS), the morphological changes related to apoptosis were assessed by fluorescence microscopy (Lecia, Bensheim, Germany). Percentage of apoptotic cells was scored on at least 500 cells.

### Cell-cycle analysis

In this experiment, cells were plated in six-well dishes. Following indicated drug treatments, cells fixed in 70% ethanol at −20°C for 16 h. After washing with PBS, cells were incubated for 30 min in the dark at room temperature with PI buffer (4 mg/ml PI, 1% Triton X-100, 0.5 mg/ml RNase A in PBS) and then filtered through a 40-mm nylon filter (Falcon, USA). The cell cycle distribution was measured for 5,000 events by a FACSCalibur flow cytometer (BD Biosciences) and data were analysed using CellQuest software (BD Biosciences).

### Annexin V/PI double staining

We also carried out experiments using the annexin V-fluorescein isothiocyanate (FITC)/PI apoptosis detection kit (Millipore) to detect the early apoptotic cells. Briefly, cells were resuspended in 100 μl 1 x binding buffer containing 10 mM HEPES/NaOH (pH 7.4), 140 mM NaCl, and 2.5 mM CaCl_2_. After the addition of FITC Annexin V (2 μg/ml) and PI (2.5 μg/ml), the cell suspension was gently mixed and then incubated for 15 min at room temperature in the dark. Afterward, 400 μl of 1 x binding buffer was added to each tube followed by flow cytometry analysis within 1 h. The FITC and PI signals were measured with a flow cytometer using the CellQuest software, and 10,000 events were acquired. Quadrants were positioned on Annexin V/PI plots to distinguish living cells (Annexin V−/PI−), early apoptotic cells (Annexin V+/PI−), late apoptotic/secondary necrotic cells (Annexin V+/PI+), and necrotic cells (Annexin V−/PI+).

### Caspase-3/7 staining

The levels of total apoptosis cells were detected by Muse Caspase-3/7 Assay Kit. 1×10^5^ cells treated for 24 h with different concentrations of Polyphyllin G. After PBS washing, prepare cell samples for incubation with Muse™ Caspase-3/7 working solution at 37°C for 30 mim. Add 150 μL of Muse™ MitoPotential 7-AAD and mix thoroughly and run on Muse™ Cell Analyzer and analysis data by the Muse®Cell Analyzer Assays (Millipore).

### Mitochondrial membrane potential (Δψm) measurement

The loss of Δψm was detected by JC-1 staining or Muse Mitopotential Assay Kit. For JC-1, after being subjected to indicate treatment, cells painted on the slides and stained with JC-1 staining for 10 min, 37°C. After washing with PBS, the morphological changes related to apoptosis were assessed by fluorescence microscopy (Lecia, Bensheim, Germany). For Muse Mitopotential Assay Kit, 1×10^5^ cells treated for 24 h with different concentrations of Polyphyllin G. After PBS washing, prepare cell samples for incubation with Muse™ MitoPotential working solution at 37°C for 20 mim. Add 5 μL of Muse™ MitoPotential 7-AAD and Incubate at room temperature for 5 min. Mix thoroughly and run on Muse™ Cell Analyzer and analysis data by the Muse®Cell Analyzer Assays (Millipore).

### In situ immunofluorescence assay

Cells were seeded into 6-well dish at a density of 4 × 10^5^ cells per dish. After Polyphyllin G incubation, cells were fixed with 4% paraformaldehyde for 20 min and then incubated with 0.5% Triton X-100 for 10 min. PBS washing was conducted between each reaction to remove any residual solvent. Afterwards, fixed cells were incubated with 4% BSA at room temperature for 2 h and then with the appropriate primary antibodies at 4°C for overnight. After overnight incubation, cells were washed and then incubated with Alexa Fluor 488-conjugated affinipure goat anti-rabbit IgG secondary antibody (Jackson Immuno Research, West Grove, PA, USA) with light protection for 1 h. At the end of incubation, cells were observed under fluorescence microscopy equipped with filters for UV and Blue 488 nm.

### Detection of acidic vesicular organelle (AVO) formation

Polyphyllin G-treated cells were washed with PBS, followed by staining with 1 μg/ml acridine orange for 30 min, 37°C. Afterwards, cells were washed with PBS and then observed under fluorescence microscopy equipped with filters for 488 nm. For quantification of AVOs, acridine orange-stained cells were harvested, washed twice with PBS, resuspended in PBS containing 5% FBS and then analyzed by flow cytometry.

### Western blot analysis

Cells were lysed in RIPA buffer containing protease inhibitor cocktail and phosphatase inhibitor cocktail. Protein concentration was determined by the BCA assay (Pierce). The equal amount of total protein was resolved by SDS-PAGE and transferred to PVDF membranes (Millipore, Bedford, MA). Membranes were blocked with 5% non-fat milk in PBS for 1 h and then incubated at 37°C for 1 h or at 4°C overnight with the indicated primary antibodies. Membranes were washed with PBS/0.1% Tween 20 (PBST) and incubated for 1 h at room temperature with the appropriate secondary antibodies conjugated to horseradish peroxidase. Membranes were then washed and bound antibodies were visualized by ECL detection system and relative photographic density was quantitated by a geldocumentation and analysis (AlphaImager 2000, Alpha InnotechCorporation, San Lean 189 dro, CA).

### In vivo anti-tumor growth effects on xenograft transplantation

As previously described [45]. For experimental xenograft growth inhibition study, 5-6 week male C57BL/6 mice (18–22 g) (National Taiwan University Animal Center, Taiwan) were used. NPC-039 cells (2×10^6^ per mouse) were resuspended in 200 μl of sterile PBS and injected s.c. into the right flank of the mouse. Mice were randomized into two groups (5 mice/group). All animals were housed with a regular 12-h light/12-h dark cycle and water ad libitum access to standard rodent chow diet (LaboratoryRodent Diet 5001, LabDiet, St. Louis, MO), and kept in a pathogen-free environment at the Laboratory Animal Unit (temperature 22°C, humidity 30~70%, 5 mice/cage). Seven days after tumor cell injection, the mice were orally fed Polyphyllin G (2 mg/kg and 4 mg/kg) or vehicle three times per week. The control group received an equal volume of 0.5% carboxymethyl cellulose vehicle. Tumor volumes were determined from caliper measurements obtained every six days. At the end of the experiment, mice were animals were sacrificed and primary tumors were removed for further analysis. The primary tumors were separated from the surrounding muscles and dermis, and then weighed. The tumor volume was calculated by the following formula: 0.5 x length x width^2^. Mean weight of mice at initiation of study and termination of study did not differ significantly between the groups. All of the procedures involving animals were conducted in accordance with the institutional animal welfare guidelines of the Institutional Animal Care and Use Committee (IACUC) of the Chung Shan Medical University.

### Tumor immunohistochemistry (IHC)

As previously described [46]. Paraffin embedded squamous cell carcinoma and paired non-cancerous tissue sections (4-μm) on poly-L-lysine-coated slides were deparaffinized in xylene and rehydrated in alcohol. Endogenous peroxidase activity was blocked with 3% H_2_O_2_. The antigen was retrieved by heating at 100 °C for 20 min in 10 mM citrate buffer (pH 6.0). After antigen retrieval, slides were washed with PBS and incubated with anti-Ki67 and anti-mouse immunoglobulin G (IgG) antibodies for 2 h at room temperature. After washing in PBS, slides were incubated with an horseradish peroxidase (HRP)/Fab polymer conjugate for another 30 min. The sites of peroxidase activity were visualized using 3,3'-diamino-benzidine tetrahydrochloride as a substrate. Gill Hematoxylin Solution II (MERCK, Darmstadt, Germany) was utilized as the counterstain. All specimens were deparaffinized and stained with hematoxylin and eosin (H&E) which was used as a light counterstain.

### Statistical analysis

Values represent the means ± standard deviation and the experiments were repeated at least three times. Statistical analyses were performed using the one-way analysis of variance (ANOVA) followed by Tukey's *post-hoc* test was used when more than three groups were analyzed. Data comparisons were performed with Student's *t* test (Sigma-Stat 2.0, Jandel Scientific, San Rafael, CA) when two groups were compared. In all cases, a *p* value <0.05 was considered to be statistically significant.

## SUPPLEMENTARY FIGURE


