# Supplementary material for: Polyphyllin G induce apoptosis and autophagy in human nasopharyngeal cancer cells by modulation of AKT and mitogen-activated protein kinase pathways in vitro and in vivo
Source: Oncotarget. 2016 Sep 2;7(43):70276–89. doi: 10.18632/oncotarget.11839 (PMC5342552; doi:10.18632/oncotarget.11839)
Supplement: Supplementary file 1 [file oncotarget-07-70276-s001.pdf]

Polyphyllin G induce apoptosis and autophagy in human nasopharyngeal cancer cells by modulation of AKT and mitogen-activated protein kinase pathways in vitro and in vivo

Supplementary Figure

Human Cell Line DNA Typing Report

Cell Line DNA Typing Report

Case Number:CID20160107
Report Date:08/09/2016

Mission Biotech
10F-3, No.3, Yuanchi Street
Nangang, Taipei
Taiwan 115
Tel: 886 2 26557128
Email: service@missionbio.com.tw

Sample Information：

i. Applicant Name:陳穆寬 Mu-Kuan Chen
ii. Institution:彰化基督教醫院癌症研究中心 Cancer Research Center, Changhua Christian Hospital
iii. Sample Description:HONE-1
iv. Sample type: Cell Pellet
v. Sample Received Date:07/28/2016

Allele table for the tested cell DNA

STR Locus	Repeat Numbers
D5S818	11,12
D13S317	10,12,13.3
D7S820	10,12
D16S539	9,10,11
vWA	14,16
TH01	6,7,9
Amelogenin	X
TPOX	8,12
CSF1PO	10,11
D21S11	27,30

Test Description：

CaseNumber: CID20160107
Test Date: 08/01/2016

Sample was extracted by Roche MagNA Pure Compact System.
DNA conc.=145.4 ng/μl
OD260/280 = 2.11
OD260/230 = 2.22

This test was performed by using the PromegaGenePrint® 10 System and analyzed by ABI PRISM 3730 GENETIC ANALYZER and GeneMapper® Software V3.7.

Verified by:
Laboratory Director(Title)

Liang Kuei Chang

08/09/2016

Human Cell Line DNA Typing Report

Cell Line DNA Typing Report

Mission Biotech
10F-3, No.3, Yuanchi Street
Nangang, Taipei
Taiwan 115
Tel: 886 2 26557128
Email: service@missionbio.com.tw

Case Number:CID20160106
Report Date:08/09/2016

Sample Information：

i. Applicant Name:陳穆寬 Mu-Kuan Chen
ii. Institution:彰化基督教醫院癌症研究中心 Cancer Research Center, Changhua Christian Hospital
iii. Sample Description:NPC-039(NPC-TW01)
iv. Sample type: Cell Pellet
v. Sample Received Date:07/28/2016

Allele table for the tested cell DNA

STR Locus	Repeat Numbers
D5S818	11,12
D13S317	10,13.3
D7S820	10,12
D16S539	9,10
vWA	14,16
TH01	6,7,9
Amelogenin	X
TPOX	8,12
CSF1PO	10,11
D21S11	27,30

Test Description：

CaseNumber: CID20160106
Test Date: 08/01/2016

Sample was extracted by Roche MagNA Pure Compact System.
DNA conc.= 76.4 ng/µl
OD260/280 = 2.11
OD260/230 = 2.06

This test was performed by using the PromegaGenePrint® 10 System and analyzed by ABI PRISM 3730 GENETIC ANALYZER and GeneMapper® Software V3.7.

Verified by:
Laboratory Director(Title)

Liang Kuei Chang

08/09/2016

1

Supplementary Data 1
